# Misdiagnosis of hepatic cystic echinococcosis complicated with hepatocellular carcinoma: A case report

**DOI:** 10.1097/MD.0000000000032291

**Published:** 2022-12-23

**Authors:** Zheng Wang, Jin-Yu Yang, Pan Xia, Hai-Hong Zhu, Zhi-Gang Gai

**Affiliations:** a Department of Graduate School, Qinghai University, Xining, Qinghai Province, China; b Department of General Surgery, Qinghai Provincial People’s Hospital, Xining, Qinghai Province, China.

**Keywords:** case report, hepatic cystic echinococcosis, hepatocellular carcinoma

## Abstract

Hepatic cystic echinococcosis (CE) is a common zoonotic parasitic disease caused by the entry of *Echinococcus granulosus* eggs into human body. Surgical resection is the optimal treatment choice for hepatic CE. However, Coexistence of CE and hepatocellular carcinoma (HCC) have been reported with a rare incidence rate, which led to unsatisfactory prognosis after the operation.

**Patient concerns:**

A 69-year-old male patient was admitted to hospital because of “Upper abdominal pain and discomfort for more than 1 month and an aggravation for 10 days.”

**Diagnosis:**

An elderly male herder who was initially diagnosed as hepatic CE, and none of the preoperative imaging test revealed the existence of HCC. Co-existence of hepatic CE and HCC was confirmed by the postoperative pathological examination.

**Interventions:**

The patient underwent “combined hepatic segmental resection, portal vein thrombectomy, portal vein repairment, hepatic hydatid internal capsule removal and external subtotal resection, cholecystectomy”.

**Outcomes:**

During follow-up after discharge, the patient did not regularly review and get further treatment and died 8 months after operation.

**Lessons:**

May improve the clinicians’ understanding of CE complicated with HCC, and reduce the misdiagnosis of similar case, as well as provide guidance for clinical treatment.

## 1. Introduction

Cystic echinococcosis (CE) is a globally endemic zoonosis caused by the larval cyst stage of the dog tapeworm *Echinococcus granulosus*. Liver is the most targeted organ for CE, and surgical resection is the optimal curative treatment for hepatic CE. Hepatocellular carcinoma (HCC) is the most common primary liver cancer, with high morbidity and mortality.^[1,2]^ The 2 major causes of liver cancer are chronic hepatitis B virus (HBV) and hepatitis C virus infection, and other risk factors including nonalcoholic fatty liver disease and the abuse of alcohol and tobacco also accelerate the development of liver cancer.^[3]^

Up to date, hepatic CE combined with HCC has been rarely reported. The association between these 2 diseases is still not well-defined.^[4]^ In this case report, we report hepatic CE with HCC in an elderly male herder, and none of our preoperative imaging studies revealed the existence of HCC, we only confirmed it in the postoperative pathological findings. Our case report may improve the clinicians’ understanding of CE complicated with HCC, and reduce the misdiagnosis of similar case, as well as provide guidance for clinical treatment.

## 2. Case report

A 69-year-old male patient was admitted to Qinghai Provincial People’s Hospital on November 24, 2021 because of “Upper abdominal pain and discomfort for more than 1 month and an aggravation for 10 days.” One month before admission, the patient suffered from paroxysmal upper abdominal pain without obvious inducement and nausea without vomiting. The patient did not pay attention to it at that time and did not receive any relevant diagnosis and treatment. Ten days ago, the pain gradually worsened, and the patient then got a low fever, cough and sputum. The abdominal ultrasound examination from local hospital revealed a space-occupying lesion in the liver, which was considered as hepatic hydatid disease, and the patient was suggested for further treatment in superior hospital. The patient denied HBV infection, and he has a history of life in a pastoral area for about 60 years. The patient underwent surgery for hepatic echinococcosis 10 years ago. Physical examination revealed mild pressure pain in the right upper abdomen, no significant rebound pain and muscle tension.

## 3. Examination results

**Figure 1.**
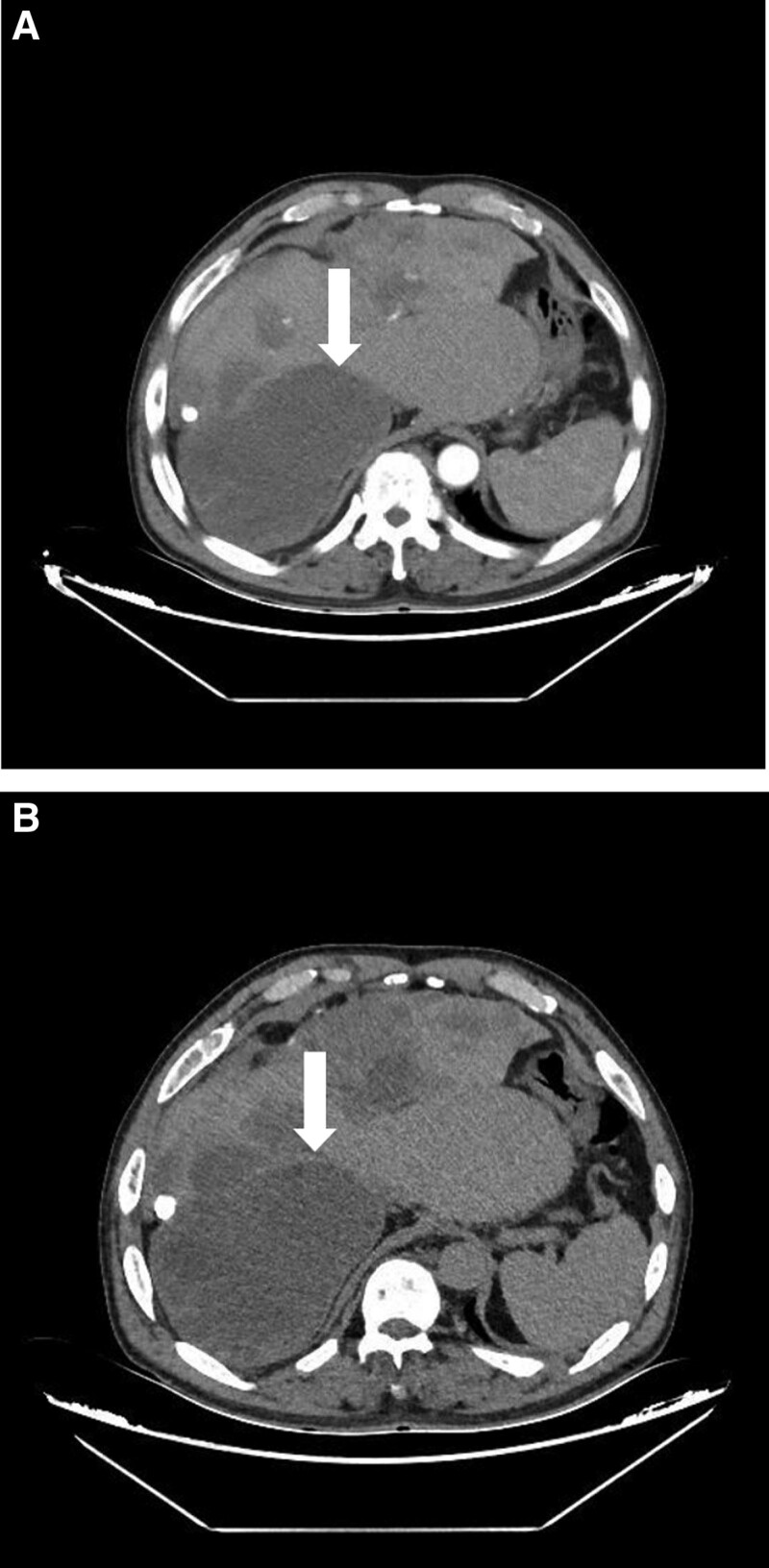
(A and B) In the arterial phase and delayed phase multiple mixed density shadows were found in the right lobe of liver, and no enhancement was found on enhanced scan (white arrow).

**Figure 2.**
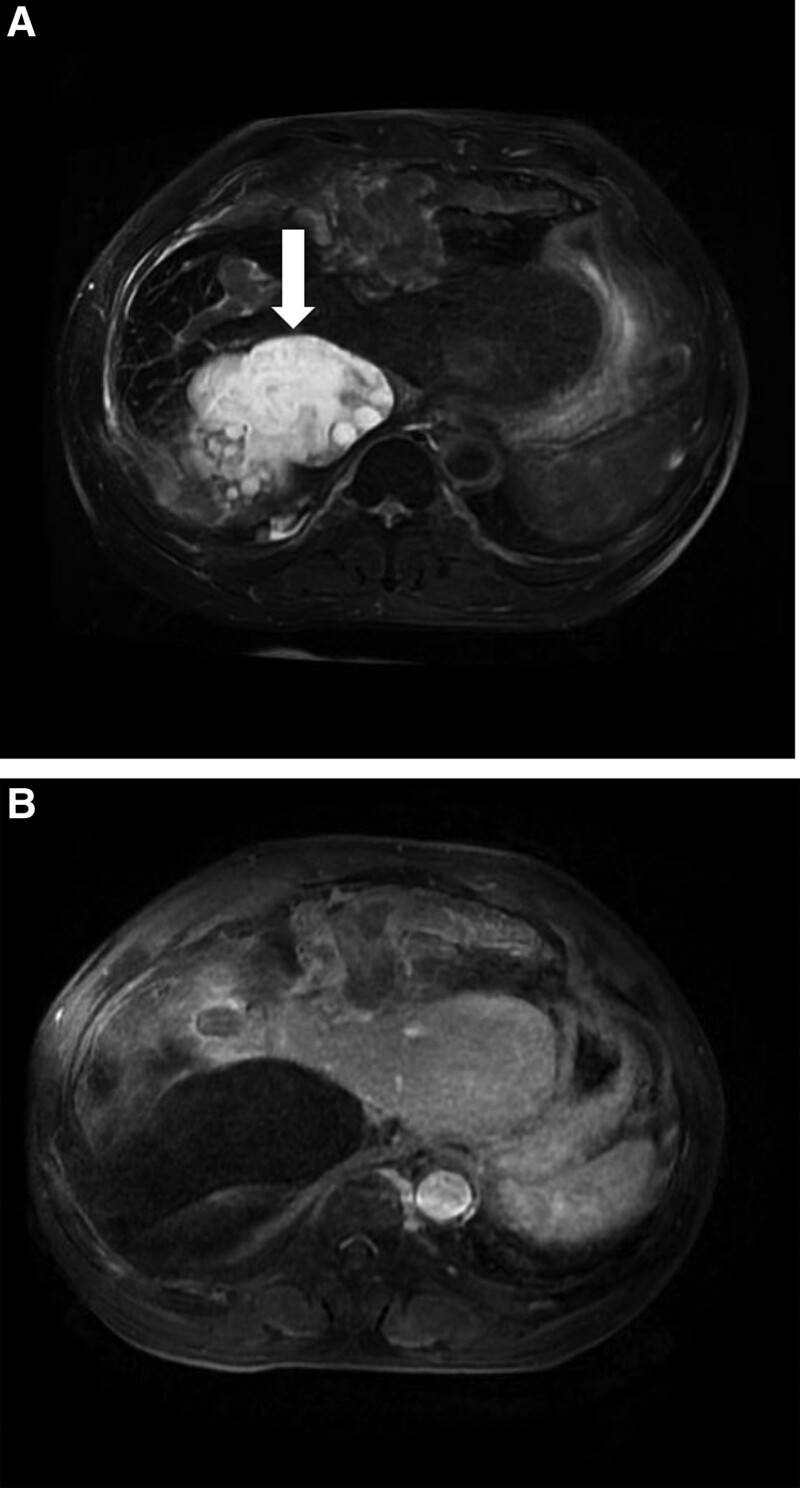
(A and B) Preoperative abdominal magnetic resonance imaging (MRI) images (the white arrow direct the large cystic hydatide of the right lobe of the liver).

The patient’s laboratory test results in (Table 1). And the computed tomography (CT) examination showed that there were multiple mixed density shadows in the right lobe of liver and abdominal cavity without enhancement at the arterial phase and delayed phase (Fig. 1A and B).The magnetic resonance imaging (MRI) examination revealed that liver edge showed smooth, partial liver surface were uneven, and multiple round-like long T1 mixed with T2 abnormalities were found in the right lobe of the liver and the hepatogastric space with no significant enhancement. The larger one was located in the right lobe of the liver, with a maximum cross-sectional size of approximately 121 × 117 mm (Fig. 2A and B).On the basis of the above-mentioned imaging findings, the patient was only diagnosed with hepatic echinococcosis preoperatively and underwent the relevant surgery.

**Table 1:** The patient’s laboratory test results.

Laboratory index	Results	Reference value
WBC	5.16 × 10^9^/L	3.50–9.50 × 10^9^/L
ALT	61 U/L	9–60 U/L
AST	62 U/L	15–45 U/L
GGT	291.0 U/L	10–60 U/L
TBIL	19.5 µmol/L	5.0–21.0 µmol/L
DBIL	6.1 µmol/L	0–3.4 µmol/L
AFP	9.88 ng/mL	0–7 ng/mL
HBsAg	0.02 IU/mL	<0.05 IU/mL
HCV	0.00 COI	<1.0 COI
ELISA	4.47	<0.9

AFP = alphafetoprotein, ALT = alanine aminotransferase, AST = aspartate aminotransferase, DBIL = direct bilirubin, ELISA = enzyme-linked immunosorbent assay, GGT = gamma glutamyl transferase, HBsAg = hepatitis B surface antigen, HCV = hepatitis C virus, TBIL = total bilirubin, WBC = white blood count.

## 4. Treatment and follow-up

**Figure 3.**
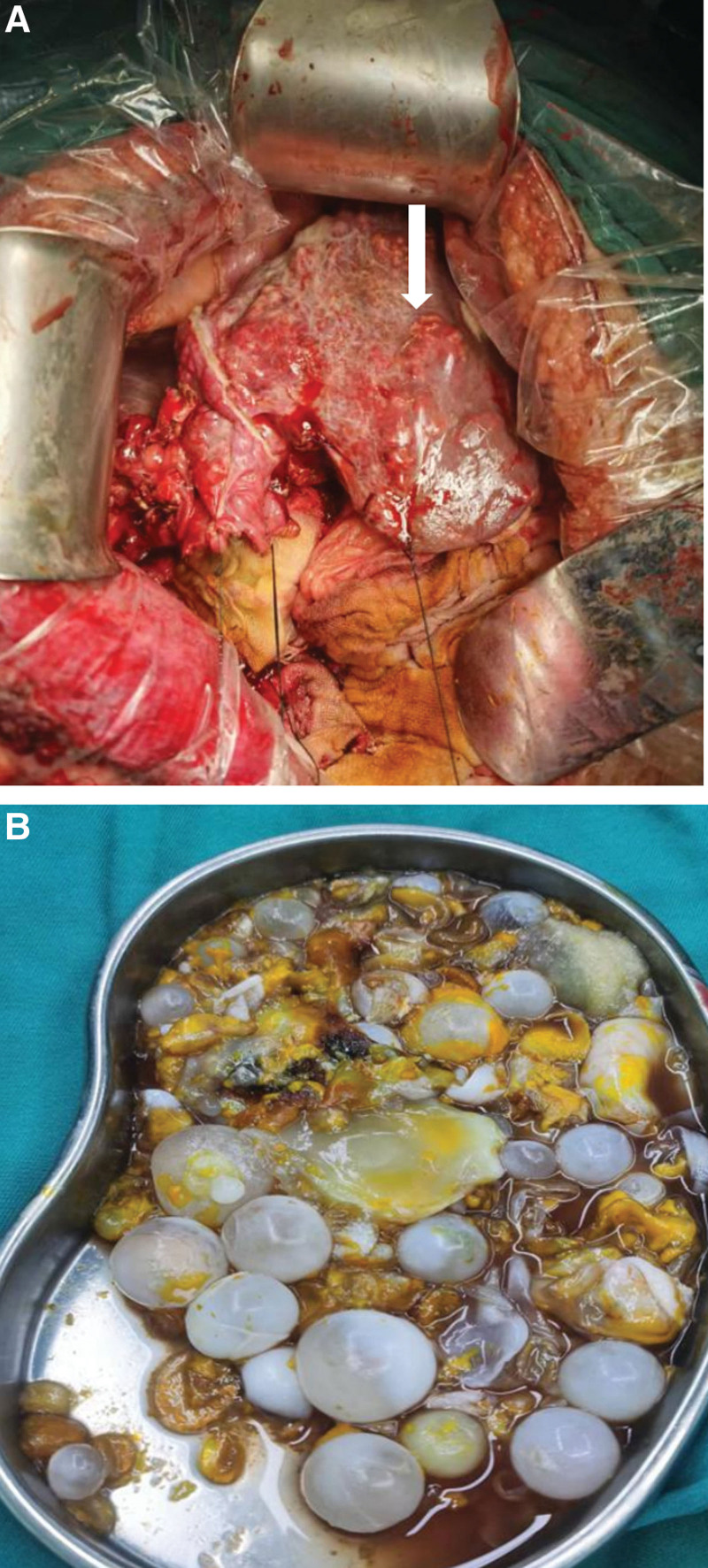
(A) Intraoperatively we see nodules of varying sizes on the surface of the liver (white arrow). (B) Internal capsule and cyst fluid of hepatic cystic hydatid.

**Figure 4.**
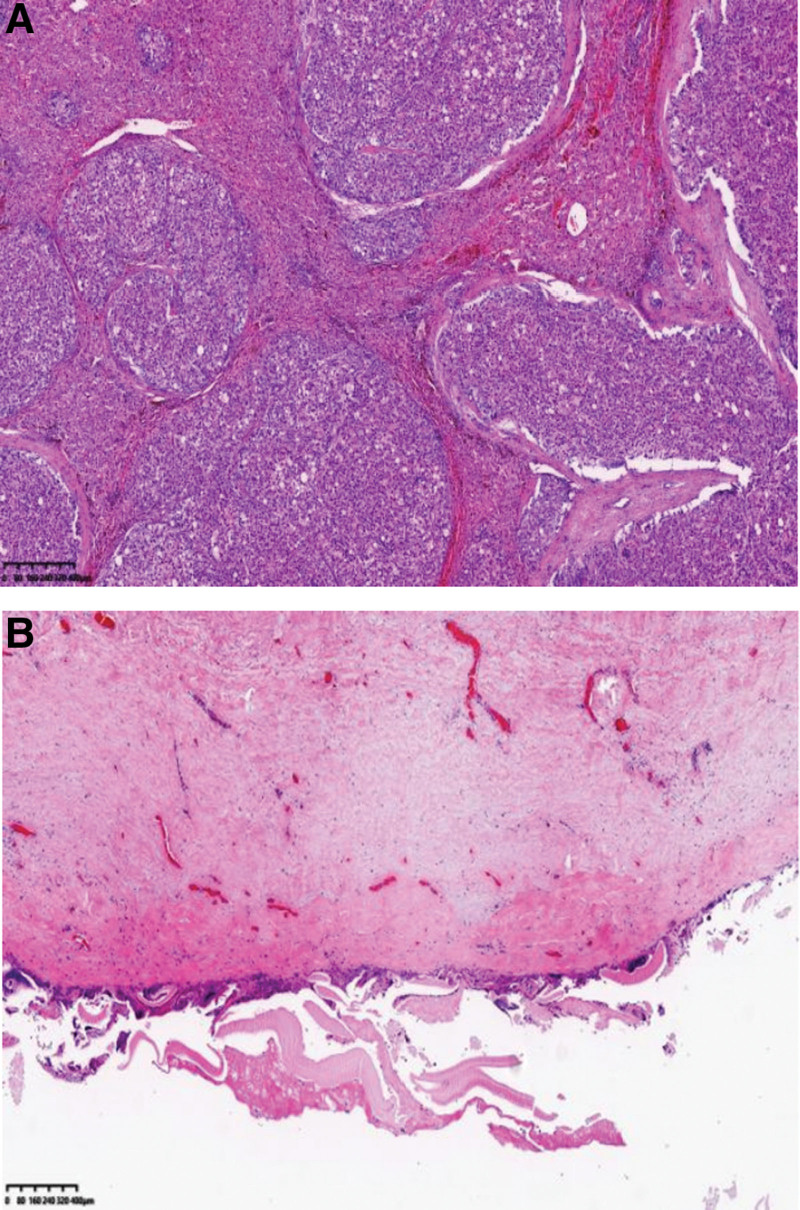
Histopathological findings (A) moderately differentiated hepatocellular carcinoma. (B) Hepatic echinococcosis infection.

The surgery of “combined hepatic segmental resection, portal vein thrombectomy, portal vein repairment, hepatic hydatid internal capsule removal and external subtotal resection, cholecystectomy” was performed under general anesthesia on December 9, 2021. During surgery, we saw nodules of various sizes on the surface of liver with reduced volume, which we considered as multiple alveolar echinococcosis in the left lobe of the liver (but we confirmed it to be HCC in the postoperative pathology). The nodules were white, hard, and uneven, and the maximum of them (7.0 × 5.0 × 4.0 cm) was in the S3 and S4 segments of the liver. There were multiple hepatic cystic hydatid (the largest was about 20.0 × 12.0 × 10.0 cm) in the right lobe of the liver, with a large number of emboli in widened portal vein (we thought it was a worm embolus in palpation intraoperatively, but postoperative pathology confirmed it was a cancer embolus). Therefore, the surgery of portal vein thrombectomy, irregular resection of S3 and S4 segments of the liver, hepatic hydatid internal capsule removal and external subtotal resection of the right lobe of the liver were performed (Fig. 3A and B). Unexpectedly, postoperative pathology showed that the specimen was a collision of 2 lesions: moderately differentiated HCC and hepatic echinococcosis infection (Fig. 4A and B). However, combined with the intraoperative situation, we considered that the patient had multiple nodular HCC of the liver, which also invaded the portal vein and the first hepatic hilar, and had lost the chance for radical surgery. We recommended further treatment in the medical oncology department 3 to 4 weeks after discharge. During telephone follow-up after discharge, the patient did not regularly review and get further treatment and died 8 months after operation.

## 5. Discussion

Hepatic CE is widely distributed around the world in areas with developed agriculture and animal husbandry, and most commonly settles in the liver.^[5]^ Various treatments like drug therapy, puncture, aspiration, injection, and reaspiration are applied to clinical practice, but the traditional surgical resection is still the main treatment. Although hepatic CE is relatively common in clinical practice, CE combined with liver cancer is rare in clinical practice, especially for some atypical cases, which is more difficult to diagnose. The patient mentioned in this article had no previous history of HBV infection and no significant elevation of alphafetoprotein (AFP) on admission tests. Neither CT nor MRI suggested HCC preoperatively, and intraoperatively we mistook the nodular HCC for alveolar echinococcosis. Ran Bo^[6]^ mentioned that CE and HCC are both chronic diseases without typical clinical manifestations, whereas CE lesions can exhibit tumor-like infiltrative growth features in the liver through extensive proliferation of metazoan bodies, making identification of CE with HCC more difficult in many clinical situations. Therefore, physicians should be more cautious in the differential diagnosis of CE and HCC in the future.

Tatsuo Kanda^[7]^ noted that HCC is mainly caused by viral hepatitis and about 70% of HCC patients have HBV or hepatitis C virus infection. Ping Luo^[2]^ thought that AFP is considered to be a widely used biological marker for HCC. However, as we reported that the patient had no previous HBV infection and the AFP was only mildly elevated in the preoperative examination, we believe that the cause of the patient’s HCC may be the compression of the surrounding liver tissues by hepatic cysticercosis, which then caused inflammatory changes around the liver and eventually cirrhosis, leading to an increased risk of developing HCC, which is consistent with the idea of Kübeck M.^[8]^ However, there is still lack of strong evidence to prove this conjecture. Nowadays, more and more studies have shown that echinococcosis infection may be associated with the occurrence of liver cancer.^[1,9]^ Yasen A^[9]^ revealed that *E. granulosus* protoscoleces promoted the proliferation, migration and invasion of liver cancer cells, but the mechanisms were unclear. In contrast, Berriel E^[10]^ thought the human hydatic cyst fluid could significantly inhibit colon cancer growth by inducing anti-tumor immunity. Given the paradoxical association of echinococcosis infection and cancer, more endeavor should be taken to explore the effect of each other and the specific mechanism.

Fu J^[11]^ mentioned that the diagnosis and screening for HCC are still mainly on CT, MRI, ultrasound imaging, and the level of AFP at present. Therefore, it is not difficult to diagnose typical HCC patients with relevant examination test results and history of hepatitis and cirrhosis. But in our report of this case, we missed diagnosis of the HCC preoperatively and intraoperatively due to all the above tests being negative in this patient. So we suggest that before surgery, we should ask the patient’s medical history and family history in detail, and actively explore tumor markers with strong specificity and sensitivity in liver cancer. For some patients with complex hepatic echinococcosis, positron emission tomography-CT examination can be recommended. During the operation, we should not only rely on clinical experience for some patients with uncertain diagnosis. When necessary, intraoperative frozen pathological examination should be performed to determine the surgical plan.

## 6. Conclusion

In conclusion, both CE and HCC are common in clinical practice, but the co-occurrence of both is rare. We report this case of a missed diagnosis of liver cancer and our reflection, aiming to provide guidance for such patients in future clinical work, so as to reduce the occurrence of missed diagnosis and misdiagnosis. However, the mechanism of the CE and HCC is still unclear, and more studies are needed to verify it.

## Author contributions

**Conceptualization:** Zheng Wang.

**Data curation:** Zhi-Gang Gai.

**Formal analysis:** Zheng Wang, Pan Xia.

**Funding acquisition:** Hai-Hong Zhu.

**Investigation:** Zheng Wang, Pan Xia.

**Project administration:** Hai-Hong Zhu.

**Supervision:** Hai-Hong Zhu.

**Visualization:** Zheng Wang, Pan Xia.

**Writing – original draft:** Zheng Wang, Jin-Yu Yang.

**Writing – review & editing:** Zheng Wang, Jin-Yu Yang.
